# Male age: negative impact on sperm DNA fragmentation

**DOI:** 10.18632/aging.101946

**Published:** 2019-05-14

**Authors:** Elena Albani, Stefano Castellano, Bruna Gurrieri, Luisa Arruzzolo, Luciano Negri, Elena M. Borroni, Paolo E. Levi-Setti

**Affiliations:** 1 Division of Gynecology and Reproductive Medicine, Department of Gynecology, Humanitas Fertility Center, Humanitas Research Hospital, 20089 Rozzano (Milan), Italy; 2 Humanitas Clinical and Research Center, 20089 Rozzano (Milan), Italy; 3 Department of Medical Biotechnology and Translational Medicine, University of Milan, 20090 Segrate (Milan), Italy; * Equal contribution

**Keywords:** male age, sperm, sperm DNA fragmentation (sDF), fertility, DNA Fragmentation Index (DFI), Density Gradient Centrifugation (DGC), Assisted Reproductive Techniques (ART)

## Abstract

The main goal of semen processing in Assisted Reproductive Techniques (ART) is to select sperm with good viability and, at the same time, remove Reactive Oxygen Species (ROS) sources (such as leukocytes) and reduce the percentage of morphologically abnormal sperm for fertility treatment. We performed a comparative analysis on sperm DNA fragmentation after Density Gradient Centrifugation (DGC) using products sold by two competing companies. Our results showed comparable DNA Fragmentation Index (DFI) after treatment with both DGC products. However, in both cases, a comparable number of samples do not benefit from the treatment. Interestingly, increasing evidences indicated that male age has a negative impact on sperm DNA fragmentation, but the mechanisms underlying age-dependent patterns of sperm decline have not yet been fully understood. Thus, we performed a comparative analysis of DFI before and after treatment with DGC products in age-stratified sample populations. Our results showed a worsening of the baseline DFI in the eldest group and the benefits of DGC on sperm DNA were compromised. In conclusion, our work consolidates the current evidences suggesting that both paternal and maternal aging, critically affects reproductive success.

## Introduction

In somatic cells’ nuclei DNA molecules spiralize around histone proteins, thus reducing their volume; on the contrary, in spermatozoa, during the final post-meiotic phases of spermatogenesis, histones are replaced with protamines. This step represents a specific mechanism of epigenetic control and allows for obtaining an additional compaction degree of sperm DNA to intensify its resistance to external damages [1,2]; yet, this mechanism becomes less efficient with aging, weighing on fertility [3]. In fact, sperm DNA Fragmentation (sDF) is strictly associated with failure and/or longer time to conceive, impaired embryo development and higher miscarriage rates [4–8].

The exact mechanism for age-dependent patterns of sperm decline is still not fully understood. Several factors, such as the free radical theory or changes in telomerase, have been discussed in literature [9]. Oxidative stress is one of the main factors triggering sDF and it occurs when there is no balance between ROS concentration, which is required for many cell pathways, and antioxidant defenses. Such a condition is common in many disease states and leads to male infertility [10–13]. Indeed, high ROS concentrations, as observed during aging, cause damages to cell components, such as lipids and proteins [9,14]. Lipid peroxidation occurs when the double bonds of an unsaturated fatty acid are attacked by a free radical, creating therefore a lipid peroxide radical. The final result of this process is a self-propagating reaction which causes marked damages to lipid membranes, thus affecting membrane fluidity. In addition, the products of lipid peroxidation are both mutagenic and genotoxic to DNA [15,16]. Spermatozoa are particularly vulnerable to lipid peroxidation, as they contain high concentrations of docosahexaenoic acid (an unsaturated fatty acid with six double bonds per molecule, which triggers ROS generation, leading to compromised motility) [17–19].

The main goal of semen processing in ART techniques is to select sperm with good viability and, at the same time, remove ROS sources and reduce the percentage of morphologically abnormal sperm. In addition, according to WHO semen parameter guidelines, the ideal semen processing method should be the least aggressive possible, in order to minimize sperm damage and maximize the recovery of morphologically and functionally normal sperm [20,21].

This study focuses on both intrinsic causes of sperm DNA injuries, including male aging, and extrinsic ones, including treatments that semen undergoes in the laboratory. The understanding of these causes may therefore help technicians prevent such effects. Up to now, Swim-Up (SU) and DGC remain the most commonly used semen preparation methods for assisted conception. It is however believed that DGC may cause an increase in ROS concentration, which as before stated, could lead in turn to DNA damages. According to WHO guidelines, the SU method is useful in selecting motile spermatozoa as it is based on the ability of sperm to swim into the culture medium, while DGC separates the various cell types [21]. The effects of these main semen preparation methods, in terms of reducing the percentage of sDF, appear to be different depending on the method used to assess sDF and the study participants. However, in scientific literature there is a certain discrepancy regarding this matter. On the one hand some studies report that DGC preparations are indeed better than SU in terms of reducing the proportion of sperm DNA fragmentation. On the other hand, there are also studies reporting that the two have similar efficacy [22,23]. Nonetheless, most recent literature states that in general any kind of sperm separation can be beneficial in terms of reducing DNA fragmentation [24].

In our study we used two different DGC products. We then analyzed the different effects of these products on semen samples. Our analysis was focused on determining sDF levels before and after treatment, splitting the sample populations in two age-dependent groups, and our final goal was to ultimately improve semen processing based on research results.

## RESULTS

### Effects of DGCs on semen parameters

Semen parameters of sperm samples were analyzed before and after DGC treatments. As shown in Table 1, no statistically significant differences in sperm concentrations, progressive and hyperactivated motility were observed between samples treated with either PureSperm® or Gradient™.

**Table 1:** Effects of DGCs on semen parameters.

**Semen parameters**	**Mean (±SD)**	***P* value**
PureSperm® concentration (×10^6^/mL) Gradient™ concentration (×10^6^/mL)	27.19 (±15.52) 25.51 (±16.57)	0.1062
		
PureSperm® PM (%) Gradient™ PM (%)	73.53 (±8.46) 72.07 (±8.07)	0.0629
		
PureSperm® HM (%) Gradient™ HM (%)	42.86 (±17.10) 45.61 (±18.58)	0.0635

Parameters of processed samples, expressed as mean±SD. PM: progressive motility, HM: hyperactivated motility. P value was obtained thanks to Student’s t test and refers to PureSperm® versus Gradient™.

### Effects of DGCs on DFI

**Figure 1:**
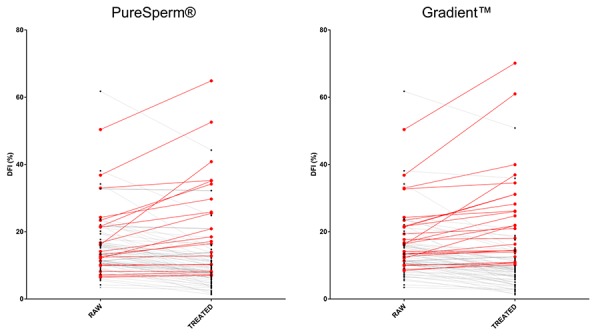
**Effects of DGCs on single sample DFI.** Graphs showed DFI values before and after DGC treatments. PS: PureSperm®; GD: Gradient™. Black and red dots/lines stand for samples showing decreased and increased DFI values after the indicated treatments, respectively.

DFI values of untreated and DGC-treated sperm samples were analyzed in Table 2. Results showed that baseline DFI values were significantly improved after PureSperm® (68/89 samples: 76.40%) and Gradient™ (64/89 samples: 71.91%) DCG treatments likewise. However, single samples analysis revealed that a significant proportion of sperm samples did not benefit from DCG treatments (30/89 samples: 33.70%) (Figure 1). A detailed analysis of single sample DFI trend before and after DGC treatments has been shown in Supplementary Table.

**Table 2:** Effects of DGCs on DFI.

**DFI (%)**	**Mean (±SD)**	***P* intergroup**
A: Raw semen	15.65 (±9.32)	A vs. B ****
B: PureSperm® DGC	12.97 (±11.27)	A vs. C ****
C: Gradient™ DGC	13.83 (±12.30)	B vs. C ns

DFI values of the 3 fractions analyzed in this study, expressed as mean±SD. ****: *P*<0.0001; ns: not significant. *P* value was obtained thanks to One-way ANOVA and the post hoc analysis (Tukey’s test).

### Effect of age on semen parameters

In order to determine the factors responsible for the increased DFI in the small cohort of sperm samples previously identified, we stratified our sample population into two age-groups (Group A: 19-38 and Group B: 39-51 years old) as indicated in detail in Materials and Methods section. Analysis of semen parameters was shown in Table 3. Results showed no differences between the groups. Although our results would seem to thwart data in literature that clearly showed a negative effect of age on semen quality [2,25,26], they are expected as our experimental design included only a cohort of samples with good baseline seminal parameters, according to WHO guidelines (see details in Materials and Methods section). Indeed, our experimental strategy aimed at emphasizing male age as the only parameter responsible for increased DFI.

**Table 3:** Effect of age on semen parameters in age-stratified samples.

**Semen parameters**	**Group A** **Mean (±SD)**	**Group B** **Mean (±SD)**
Age	34 (±3.67)	42.89 (±3.37)
Ejaculate volume (mL)	3.77 (±1.07)	3.41 (±0.91)
Concentration (×10^6^/mL)	60.46 (±19.99)	63.87 (±21.71)
Progressive motility (%)	30.08 (±6.32)	29.12 (±6.29)
Viability (%)	78.1 (±5.63)	77.35 (±7.02)

Parameters of unprocessed samples of Group A and Group B expressed as mean±SD.

### Effects of age on raw and DGC-treated DFI

**Figure 2:**
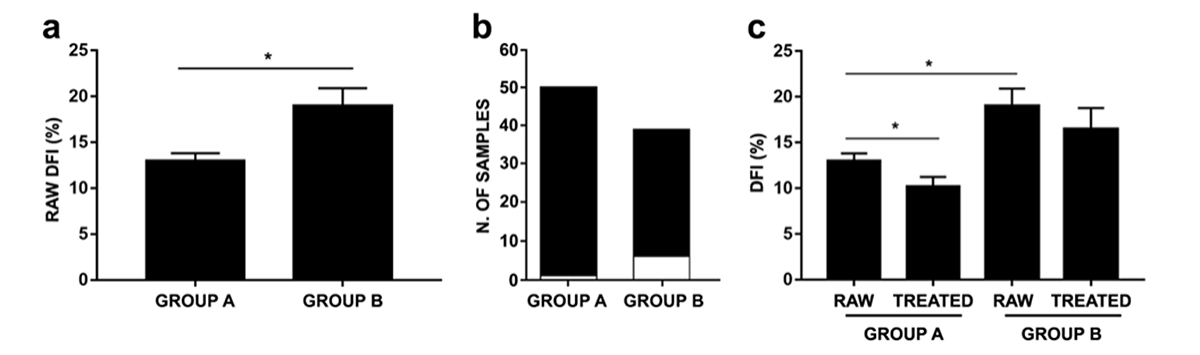
**Effects of age on raw and DGC-treated DFI in age-stratified samples.** (**a**) Raw DFIs belonging to Groups A and B (P=0.0122). (**b**) Number of samples with DFI <30% (black bar) and >30% (white bar) within Group A and B; (**c**) Raw and treated DFIs belonging to Groups A and B (group A: *P*=0.0007; group B: *P*=0.0692). As PureSperm® and Gradient™ DGC treatments showed comparable efficacy, analysis has been carried on using PureSperm® values. Results were expressed as mean±SEM. *P* value was obtained thanks to Mann-Whitney test.

DFI values of untreated and DGC-treated sperm samples were analyzed in both groups. As shown in Figure 2a, raw DFI of Group B showed a significant increase compared to Group A. Moreover, a single sample analysis revealed that more samples from Group B (6/39 samples: 15.38%) showed a higher baseline sDF exceeding the SCSA threshold level (>30% as indicated in Materials and Methods section) compared to Group A (1/50 samples: 2%) (Figure 2b). Interestingly, whereas no statistical difference between raw and treated DFIs of Group B were observed, treated DFIs of Group A were further increased compared to baseline (Figure 2c).

## DISCUSSION

The increasing demand for assisted reproductive technologies has led to necessary improvements in the procedures routinely used in *in vitro* fertilization (IVF) laboratories [27,28]. Several methods are currently available and commonly used in the field of semen preparation, but there is no consensus on which method is most efficient Each of these techniques, based on either sperm migration or separation, is beneficial in selecting a morphologically normal spermatozoa population, characterized by a valuable progressive motility. Nevertheless, these are not feasible techniques for separating cells with and without DNA fragmentation within the sperm population, despite fragmentation being correlated to other measurable parameters (e.g. progressive motility) [29]. Different techniques are available for the assessment of DNA fragmentation but none of them can be used clinically, due to the fact that once tested, the semen can no longer be used [24]. In this study we chose to select sperm with DGC techniques, comparing the efficiency of two different brands [PureSperm® 100 (*Nidacon International*, Gothenburg, Sweden) and Gradient™ 100 (*Origio®*, Stenløse, Denmark)] to raw semen (the comparison parameter was DFI).

Our study shows how DGC selection does not always safeguard from DNA injuries. Indeed, before stratifying by age, we observed that in 23.59% of samples (21/89) treated with PureSperm® and in 28.08% of samples (25/89) treated with Gradient®, post-DGC sDF rate was higher when compared to pre-DGC values, suggesting the induction of a *de novo* DNA damage during the procedure. Although initial studies suggested that the shearing forces generated during centrifugation can damage sperm DNA by generating ROS, recent data points out that contamination of colloidal silicon gradients with transition metals is the main cause of oxidative stress and breakage to sperm DNA whilst going through density gradient centrifugation techniques [30–32]. However, in this study most of the samples are subjected to a decrease in sDF (76.41% for PureSperm® and 71.91% for Gradient™), indicating that metal contamination is not enough to induce the injury. As a result of our study and due to a thorough analysis of the available literature on this issue, we have come to the conclusion that defects in sperm chromatin maturation, lower sperm defenses towards oxidative attack and/or too high levels of ROS concentration in unprocessed sperm may result in an increased sensitivity of the semen to corruptive agents [26,33].

Correlation between aging and sperm DNA integrity represents another controversial factor. As men undergoing ARTs grow older, testicular function and metabolism deteriorate due to the testis’ age-related morphological changes, such as the decrease in the number of germ cells, Leydig and Sertoli cells, as well as structural changes, including the narrowing of seminiferous tubules [34,35]. Throughout the course of aging, the regulation of hypothalamic-pituitary-gonadal axis is altered and apoptosis or accumulation of ROS in male germ cells can occur. These events may lead to oxidative stress and insults to sperm DNA [36]. In our study we chose to split the entire population of samples into two age-dependent groups, in order to check the development of sDF in age-differentiated populations. As shown in Figure 2a, group A exhibited lower raw DFI levels than group B, indeed 15.38% of the samples (6/39) belonging to the latter showed a raw sDF exceeding the SCSA threshold level; what happened in group A was clearly different: only 2% of the samples (1/50) exceeded the sDF threshold level in untreated semen. After stratification, we noticed how the treatment (either PureSperm® and Gradient™ DGC) lowered the DFI rate of the samples belonging to group A, while it did not have a significant effect on group B samples DFIs. Moreover, several studies report significant correlations between male aging and sperm DNA fragmentation [37–41]. As homogenous and copious clinical records concerning the two study groups analyzed were missing, we avoided correlation test. Furthermore, we analyzed no men above 51 years old, due to the difficulty in obtaining samples from men with advanced age, defined by the inclusion criteria we chose for this study. The scientific literature shows indeed how complex it is to realize a study concerning the issue of ART, taking into consideration a significant number of individuals belonging to the elderly population (above 60 years of age). Moreover, another confounding factor is the lack of data related to internal or andrological pathologies or Body Mass Index (BMI), which may be causal factors of alteration of seminal characteristics. This concept is in accordance with clinical andrological experience, which shows that a fast track to recovery of male fertility can be achieved with early diagnosis and treatment of several andrological pathologies [42]. Whereas the results concerning the effects of DGCs on sDF rates are solid (performed on 89 samples), it is still necessary to enlarge the number of recruited men in the future and to consider as well independent variables, so as to achieve statistically significant as much as clinically relevant results.

Our study points out that DGC selection for ART can hinder the DNA integrity, as seen in 33.70% of the total of our samples (30/89 samples), therefore implying a possible risk for pregnancy. In this percentage of the population we should investigate which other factors could negatively influence the DFI of samples undergoing DGC treatments. These findings show how gradient preparation procedures currently in use to select sperm should be improved or that an alternative way to better treat those samples should be found, in order to avoid DNA damage due to density centrifugation, particularly in patients greater than 38 years of age. Research on this subject matter should be broadened in the future, in order to obtain a more detailed analysis based on a more consistent number of stratified samples per age.

Though our study’s main focus is the effect of ART treatments on semen, through the analysis of the available data and the results of our study itself, we have come to the conclusion that factors which were previously considered of minor importance, are actually of utmost significance. In particular, the stereotypical concept of the “biologic clock”, which in the past was applied only to female individuals, has indeed a great impact on men’s fecundity. This recent finding may therefore have a clinical implication, meaning that the main factors determining infertility of the couple as a whole should be reevaluated and that the age of the male partner should be taken into account, at least as much as that of the female.

## MATERIALS AND METHODS

### Sample inclusion criteria

Semen samples were obtained by masturbation by 89 men aged 19 to 51. Between January and June 2018, the semen underwent further diagnostic analysis at the Humanitas Fertility Center, Department of Gynecology and Reproductive Medicine, Humanitas Research Hospital, Rozzano (Milan), Italy. The study was approved by Independent Ethics Committee (Retrospective Study approval no. 33/18, issued on 18 December 2018) of IRCCS Istituto Clinico Humanitas (Rozzano, Milan, Italy). A written informed consent has been obtained from each patient for the use of discarded material in clinical research. Seminal parameters were chosen mainly to obtain at least the minimum concentration necessary for Sperm Chromatin Structure Assay (SCSA) (2×10^6^ spermatozoa/mL). From this point of view, it’s noteworthy to emphasize that, most of all, the concentration and the motility in treated samples are different from those checked in fresh samples. More specifically, these parameters, assessed by a single operator, were: ≥30×10^6^ sperm concentration, ≥15% progressive motility (spermatozoa moving actively, either linearly or in a large circle, regardless of speed), ≥40% total motility, ≥58% sperm vitality and ≥2.5 mL volume, according to WHO parameters [21]. The concentration of spermatozoa was evaluated using the Makler count chamber (200x magnification). The motility was determined putting a drop on a glass slide covered by a glass coverslip, then this step was repeated on a second drop of semen prepared in the same way (400x magnification). The vitality was assessed by eosin test on a glass slide covered by a coverslip (400x magnification), detecting the proportion of living spermatozoa. The sperm volume of each sample was estimated in a 15 mL sterile centrifuge conical tube.

In our experimental design, samples were age-stratified according to data present in literature. In García-Ferreyra J. *et al.* paper, patients were split in 3 groups, choosing ≤39 years, 40 – 49 years and ≥50 years as cut-off [43]. In another research article, Colin A. and his team stratified patients in 5 year-ranges and showed how DFI increased with age, especially in over 40 years subjects [40]. Choosing 38 years as cut-off, samples were divided into two groups: Group A, characterized by 50 samples belonging to men aged between 19 to 38 years, and Group B, characterized by 39 samples belonging to men aged between 39 to 51 years.

### Sperm preparation

Semen samples were collected into sterile cups after 2-7 days of sexual abstinence. Samples were left to liquefy for a maximum period of 30 min in an incubator at 37°C. The following step was an initial evaluation of semen parameters which aimed at making sure that sperm samples respected the before mentioned parameters (see paragraph “Sample inclusion criteria”).

2 mL of each sample were divided in equal fractions and stratified in two different DGC solutions based on a colloidal silica suspension in an isotonic salt solution: PureSperm® 100 (*Nidacon International*, Gothenburg, Sweden) and Gradient™ 100 (*Origio®*, Stenløse, Denmark). The two colloidal suspensions were diluted in two sperm preparation media containing HEPES buffer to achieve 80% and 40% dilution for both DGCs: PureSperm® 100 solution was diluted in Sperm Washing Medium (*IrvineScientific®*, Santa Ana, California, USA), while Gradient™ 100 in Sperm Wash (*Origio®*, Stenløse, Denmark). Then, each gradient was obtained putting 1 mL of 80% solution on the bottom of a 15 mL sterile centrifuge conical tube and after that layering on it 1 mL of 40% solution. Then, 1 mL of semen was gently stratified above the gradients. After centrifugation at 400g for 15 min at room temperature in Centrifuge 5702 R (*eppendorf*, Leipzig, Germany), the supernatant was removed, while the pellet was resuspended in 0.5 mL of sperm preparation medium.

After the separation test, recovery sperm concentration, progressive and hyperactivated motility were evaluated by a single operator who acquired autonomy in reading the semen samples after a period of training regularly tracked in the assessment of competences by the quality control system of Humanitas Fertility Center. Hyperactivated motility was assessed subjectively by visual observation of the flagellar beat using descriptive criteria such as ‘serpentine’ and ‘figure-of-eight’ [44,45]. The fundamental movement change which occurs during hyperactivation is an increase in the amplitude of the flagellar beat caused by an increase in the bending of the proximal flagellum [45,46]. Afterwards, each sample was eventually divided in 6 smaller fractions of equal volume (2 raw fractions, 2 for PureSperm® treatment and 2 for Gradient™ treatment). Each sample was diluted in PBS to achieve a concentration of 2×10^6^ spermatozoa/mL, ideal for flow cytometry analysis. Each aliquot was stored in liquid nitrogen (-196°C) until SCSA was performed.

### Sperm Chromatin Structure Assay (SCSA)

SCSA, described for the first time in 1980, is a cytometric technique characterized by the assessment of a statistically significant number of events, as it analyzes almost 5000 spermatozoa per sample. By Acridine Orange (AO) dye, SCSA evaluates the resistance of sperm chromatin to the action of denaturing agents. When excited with 488 nm light source, AO can either emit green or red fluorescence; the previous when inserted into double stranded-DNA, the latter when inserted into single stranded DNA [47,48].

DFI is a parameter used to describe the percentage of sperm with DNA susceptible to denaturation. DFI is the ratio between red fluorescence and total fluorescence (red and green). A DFI higher than 30% indicates a sperm population with a good chromatin integrity, whereas a DFI lower than 30% indicates a sperm population with an abnormal chromatin structure [48].

Cytomics FC500 Flow Cytometer with CXP Software 2.2 (Beckman CoulterTM, Brea, California) and FCS Express 6 Flow Cytometry (De Novo SoftwareTM, Glandale, California) was used to perform SCSA. SCSA reading and DFI evaluation was performed by a second operator with certified cytofluorimetric expertise who was completely unaware of the characteristics of the samples analyzed.

### Statistical analysis

Our statistical analyses were carried out with the software GraphPad Prism v. 7.00 for Windows (*GraphPad Software, Inc.*, La Jolla, California, USA).

Since DFI data did not follow a Gaussian curve, the square root transformation was used to grant homogeneity of variances before analysis, achieving a normal data distribution. Analysis of variance (one-way ANOVA) with the Greenhouse-Geisser correction was performed in order to compare the 3 DFIs data groups (raw fraction and 2 treated fractions) to check a statistical difference. Post-hoc analysis was made with Tukey’s test to pinpoint which specific means were more significant than the others and to avoid type 1 error. Thereafter, samples were divided in two groups depending on age (19-38 age range, group A, and 39-51 age range, group B). Moreover, Mann-Whitney test was applied to compare the means of the raw DFIs in groups A and B and the raw DFIs to treated ones in each group. In order to compare DGCs semen parameters, since data followed a gaussian curve, we used Student’s t test. *P* <0.05 was considered to be statistically significant.

## SUPPLEMENTARY MATERIAL

Supplementary Table
