# Supplementary material for: Male age: negative impact on sperm DNA fragmentation
Source: Aging (Albany NY). 2019 May 14;11(9):2749–61. doi: 10.18632/aging.101946 (PMC6535069; doi:10.18632/aging.101946)
Supplement: Supplementary Table [file aging-11-101946-s001.pdf]

SUPPLEMENTARY MATERIAL

Supplemental table. DFI of raw and DGC-treated semen.

SAMPLE ID	RAW SEMEN DFI (%)	PURESPERM® DGC DFI (%)	GRADIENT™ DGC DFI (%)
TREATED SEMEN DFI > RAW SEMEN DFI (RAW SEMEN DFI >30%)			
4	32,97	35,27	39,95
7	36,82	52,59	60,99
39	50,38	64,88	70,54
84	32,75	32,25	34,51
TREATED SEMEN DFI > RAW SEMEN DFI (GRADIENT DGC > PURESPERM DGC)			
13	21,39	25,86	31,12
40	9,9	10,33	10,99
49	12,12	20,87	21,92
70	10,12	10,22	10,46
TREATED SEMEN DFI > RAW SEMEN DFI (PURESPERM DGC > GRADIENT DGC)			
16	23,41	34,19	28,25
21	16,74	25,49	24,73
29	16,13	40,84	36,92
33	13,24	16,44	16,31
37	21,61	35,14	31,15
50	14,15	18,49	14,35
63	12,64	17,12	14,54
77	24,28	29,72	26,15
73	11,31	13,92	12,57
GRADIENT DGC DFI > RAW AND PURESPERM DGC DFI			
9	21,63	20,95	26
25	17,68	16,48	18,07
41	8,76	4,83	10,11
43	13,32	7,31	13,97
52	16,43	11,42	21,84
56	19,36	11,53	20,96
59	13,52	13,5	14,39
85	8,41	7,83	10,87
PURESPERM DGC DFI > RAW AND GRADIENT DGC DFI			
34	6,48	6,96	5,73
60	6,79	7,24	2,99
64	8,15	8,21	7,11
66	12,47	12,82	8,59
78	7,17	7,94	5,91

SAMPLE ID	RAW SEMEN DFI (%)	PURESPERM® DGC DFI (%)	GRADIENT®™ DGC DFI (%)

TREATMENT IMPROVEMENT (RAW DFI > 30%)			
26	38,12	24,83	35,9
54	61,75	44,27	50,86
69	34,21	14,75	14,58
TREATMENT IMPROVEMENT (RAW DFI < 30%)			
1	16,02	8,97	8,68
2	7,87	4,15	6,84
3	21,13	18,25	18,86
5	14,37	8,04	9,24
6	16,75	12,46	11,29
8	23,31	12,15	18,45
10	15,45	10,28	8,09
11	14,03	7,06	6,67
12	10,06	5,18	7,89
14	6,61	3,67	3,97
15	22,09	3,22	3,56
17	11,22	8,16	8,9
18	11,22	8,53	6,75
19	10,68	6,41	4,2
20	23,61	13,85	15,17
22	20,16	5,87	8,44
23	11,41	9,58	9,56
24	14,07	7,41	9,05
27	9,93	8,33	8,16
28	10,71	1,61	2,55
30	16,8	12,62	13,46
31	11,31	7,84	9,1
32	15,59	11,52	11,66
35	10,36	8,48	9,78
36	24,2	15,88	20,94
38	13,43	8,78	10,52
42	10,6	8,29	9,84
44	5,9	2,49	1,67
45	5,5	1,33	2,2
46	10,61	10,56	8,73
47	11,86	4,61	7,97
48	4,2	3,4	2,95
51	15,73	11,15	9,98
53	16,02	13,57	14,76
55	13,53	6,2	6,37
57	9,23	2,06	1,31
58	15,2	7,48	8,67
61	10,41	6,23	9,49
62	10,74	7,51	6,58
65	13,54	12,4	12,12
67	10,8	6,26	5,01
68	10,77	7,53	4,23
71	11,59	3,97	4,98
72	10,76	9,38	9,2
74	13,6	6,94	7,11

75	19,43	16,11	17,56
76	15,63	7,88	11,99
79	11,48	6,2	4,29
80	8,75	3,9	3,52
81	16,06	11,01	11,34
82	23,8	9,04	14,16
83	13,18	11,31	8,31
86	10,68	5,65	6,95
87	7,4	6,79	5,04
88	16,43	9,95	7,26
89	3,43	2,75	2,72

Table listed DFI values (%) before and after DGC treatments of all semen samples analyzed. DFI were grouped according to the indicated parameters.
